# Dysregulated Expression of Long Non-Coding RNA *MINCR* and *EZH2* in Colorectal Cancer

**DOI:** 10.52547/ibj.26.1.64

**Published:** 2021-12-20

**Authors:** Sajjad Cheraghi, Hamid Asadzadeh, Gholam Reza Javadi

**Affiliations:** 1 Department of Biology, Science and Research Branch, Islamic Azad University, Tehran, Iran;; 2 Basic and Molecular Epidemiology of Gastrointestinal Disorders Research Center, Research Institute for Gastroenterology and Liver Diseases, Shahid Beheshti University of Medical Sciences, Tehran, Iran

**Keywords:** Colorectal cancer, EZH2, Long non-coding RNA

## Abstract

**Background:**

As critical regulators, lncRNAs have attracted attention from researchers for diagnostic, prognostic, and therapeutic purposes in human carcinogenesis via interfering with mRNAs such as *EZH2*. Nevertheless, the potent roles and molecular mechanisms of these RNAs in CRC are not clearly known.

**Methods:**

In this study, the tissue expressions of lncRNA *MINCR* and *EZH2* mRNA between colorectal tumors and polyps were compared with the adjacent normal tissues collected from 114 Iranian patients, using real-time PCR method. Furthermore, the correlation of the expression levels of *MINCR* and *EZH2* with other clinical parameters was evaluated.

**Results:**

The significant overexpression of *MINCR* and *EZH2* were observed in the CRC tissues compared to control tissues (*p *< 0.0001). This observation confirmed the association of these expression enhancements with the pathological stage of CRC patients.

**Conclusion:**

Our findings revealed that the expression of *MINCR* significantly alters during CRC development, and it can be identified as a potential biomarker for the detection of CRC.

## INTRODUCTION

It is widely accepted that CRC is the third commonest cancer, with an increasing incidence in the world, and is affected by genetic, epigenetic and environmental factors^[^^1^^]^. Despite significant progress in CRC therapy and diagnostic techniques so far, a large number of CRC patients are still detected in advanced stages. Moreover, recent therapies are unable to accurately decode key genes and signal cascades involved in human CRC metastasis^[^^2^^]^. The development of molecular mechanisms and investigation of new diagnostic biomarkers for screening patients can be promising approaches to CRC therapy and may result in a 90% five-year survival rate^[^^3^^,^^4^^]^.

The lncRNAs have been recognized as novel molecules possessing a crucial regulatory role in the biological procedures via interference with mRNAs, direct interaction with proteins to regulate their activities or alter their localization. These molecules also are able to affect the downstream gene expression via inhibiting the RNA polymerase^[^^5^^]^. LncRNAs and the epigenetic mechanism have been suggested to be the key regulators in CRC and can be used for diagnostic, treatment, and prognostic purposes^[^^6^^]^. Deregulation of lncRNAs has been found in several tumors, where it can act as tumor suppressor genes or oncogenes^[^^7^^]^.

*MINCR*, known as an lncRNA, has been related to the *MYC* expression in *MYC*-positive lymphomas^[^^8^^]^. *MINCR* gene is located at the chromosome 8q24.3 and is intragenic to two coding genes, *ZNF696* and *GLI4*, with 3- and 9.5-kb distances^[^^9^^]^. *MINCR* is upregulated in cancer tissues and has an association with the survival rate, cell migration, and invasion in tumor tissues^[^^9^^]^. The high expression of the *MINCR* has been correlated with the size of the tumor, node, and metastasis stage^[^^10^^]^. According to some studies, *MINCR* dysregulation could be a parameter affecting the development of the human cancers, such as gallbladder cancer and hepatocellular carcinoma^[^^11^^,^^12^^]^. Nonetheless, the exact mechanism of *MINCR* function in cancer development is still largely unknown^[^^13^^]^. 

*MINCR* could activate the polycomb repressive complexes, i.e. PRC1 and PRC2, to stimulate *EZH2* expression by targeting certain miRNAs^[^^10^^]^. The polycomb group proteins are involved in gene silencing phenomena and are highly conserved between Drosophila and humans^[^^14^^]^_. _In this regard, the PRC1 and PRC2 members can control the gene silencing via the posttranslational modification of histone proteins^[^^15^^]^. *EZH2* can serve as the catalytic subunit of PRC2. Numerous documents have demonstrated the crucial role of *EZH2* in cancer initiation, progression, metastasis, and drug resistance^[^^16^^]^. Hence, researches have focused on *EZH2* as an encouraging drug target, and several *EZH2* suppressors have ‍been developed and are undergoing clinical trials^[^^17^^]^. Inhibition of *EZH2 *has been indicated to enhance the efficacy of many anticancer medicines, which reflect the potential of the combined treatment using the *EZH2* suppressors^[^^18^^]^. 

To our knowledge, no study has been performed on the expression of lncRNA *MINCR* and its association with *EZH2 *in CRC. Hence, the expressions of lncRNA *MINCR* and *EZH2* mRNA in tumors and polyps were compared with adjacent normal tissues to investigate their possible roles in CRC progression. Additionally, the positive expression correlation of lncRNA *MINCR* and *EZH2 *mRNA with CRC were reported for the first time in the present study. 

## MATERIALS AND METHODS

**Patients and tissue samples**

Samples, including control (tumor adjacent normal tissue), polyp (hyperplastic), and tumor (adeno-carcinoma) tissues, were obtained from 114 patients. All the patients referred to the Taleghani Hospital’s Research Center for gastroenterology and liver diseases were pathologically diagnosed with CRC, from 2015 to 2017. The clinical data were gathered from all the medical records of the patients. No preoperative treatment was administered to the patients, and cases who underwent any treatment and those with other diseases were excluded. Demographic and clinic-pathological characteristics of patients are given in Table 1. All tissues were directly stored in the liquid nitrogen and maintained at 80 °C till the RNA extraction. 

**RNA extraction**

Total RNA was extracted from the miRNeasy Mini Kit (Qiagen, Germany) based on Company’s guidelines. The quality and quantity of the separated RNAs were estimated by agarose gel electrophoresis (2% agarose; Gibco/BRL USA) and spectro-photometery (Nanodrop Technologies, Wilmington, Delaware, USA). Finally, cDNA was synthesized using RevertAid RT kit (Thermo Scientific, USA) and kept at -20 °C.

**Table 1:** Demographic and clinicopathological data

Data	Number (*%*)
Gender	
**Female**	61 (53.5)
**Male**	53 (46.5)
	
Age	
**≤50**	15 (13.2)
**>50**	99 (86.8)
	
BMI (kg/m^2^)	
**18.5-24.9**	59 (51.6)
**25-29.9**	48 (42.1)
**30-35**	7 (6.1)
	
Smoking status	
**Yes**	7 (6.1)
**No**	107 (93.9)
	
Family history	
**Yes**	11 (9.6)
**No**	103 (90.4)
	
Sample location	
**Colon**	105 (92.1)
**Rectum**	9 (7.9)
	
Clinical stage	
**II**	14 (32.6)
**III**	29 (67.4)
	
Type of samples	
**Tumor tissues**	43 (37.8)
**Polyp tissues**	36 (31.6)
**Control** **tissues**	35(30.6)
	
Underlying disease	
Hypertension **Yes** **No**	
	11 (8.4) 103 (91.6)
	
IBD **Yes** **No**	10 (8.8)
	104 (91.2)

BMI, body mass index; IBD, inflammatory bowel disease

**Table 2:** The sequences of the primers utilized in our study

**Gene**	**Forward primer sequence**	**Reverse primer sequence**	**Fragment (bp)**
*MINCR*	5'-TAAAACTGGTGCGCGGGTTC-3'	5'-TCAGTCACTGCTTCATCCCA-3'	114
*EZH2*	5'-ACAGTGATAGGGAAGCAGGG-3'	5'-ACTCCACTCCACATTCTCAGG-3'	174
*Beta-globin*	5'-CCCTTCATTGACCTCAACTACATG-3'	5'-TGGGATTTCCATTGATGACAAGC-3'	117

**Real-time PCR**

The SYBR Premix Ex Taq (Takara, Japan) was used for qPCRs. The normalization of the outputs was then carried out using beta-globin expression level. The software Primer3 was used to design the primers. Table 2 shows the primers sequences utilized in our study. In the next stage, a 7900 Fast Real-Time PCR System (Applied Biosystems, Thermo Fisher Scientific, Inc. USA) was used to analyze RT-qPCR and collect the required data. Thermal cycling conditions included the initial denaturation phase (95 °C for 30 s), PCR reaction phase (40 cycles of 95 °C for 5 s and 60 °C for 34 s), and dissociation phase (95 °C for 15 s, 60 °C for 1 min, and 95 °C for 15 s). The tumor adjacent normal tissue was considered as a reference sample. In the end, the PCR products were visualized on 2% agarose gel stained with green viewer, and fold changes in the relative expressions of all the target mRNAs were computed based on the comparative 2^-ΔΔCT^ technique^[^^19^^]^.

**Statistical analyses**

The statistical analyses were performed using GraphPad Prism version 8.01 and the SPSS/PC V 26.0., Chicago, IL, USA. Student's t-test and one-way ANOVA were conducted to estimate the significant differences between two groups and multi groups, respectively. Analysis of the relationship between the expression levels and clinicopathological data was performed using χ2 or Fisher’s exact test. The descriptive analysis for quantitative data was performed using mean ± SD., and a *p value* less than 0.05 was considered statistically significant. All experiments were carried out in triplicate.

**Ethical statement**

The above-mentioned sampling protocols were approved by the Medical Ethics Committee of the Shahid Beheshti University of Medical Sciences (SBUMS), Tehran, Iran (ethical code: IRC89001357). Written informed consents were collected from each participant before inclusion in the research. 

## RESULTS

**Demographic and clinicopathological data**

This study included 114 patients (53 males [46.5%] and 61 females [53.5%]) with the age range of 30 to 75 years (the average of 60.33 years). The associated demographic and clinicopathological data, including gender, age, body mass index, smoking status, family history, sample location, clinical stages, type of samples (tumor or polyp tissues), hypertension, inflammatory bowel disease of the patients are shown in Table 1.

**Overexpression of** ***MINCR*** **and** ***EZH2*** **expression in the CRC patients**

**Fig. 1:**
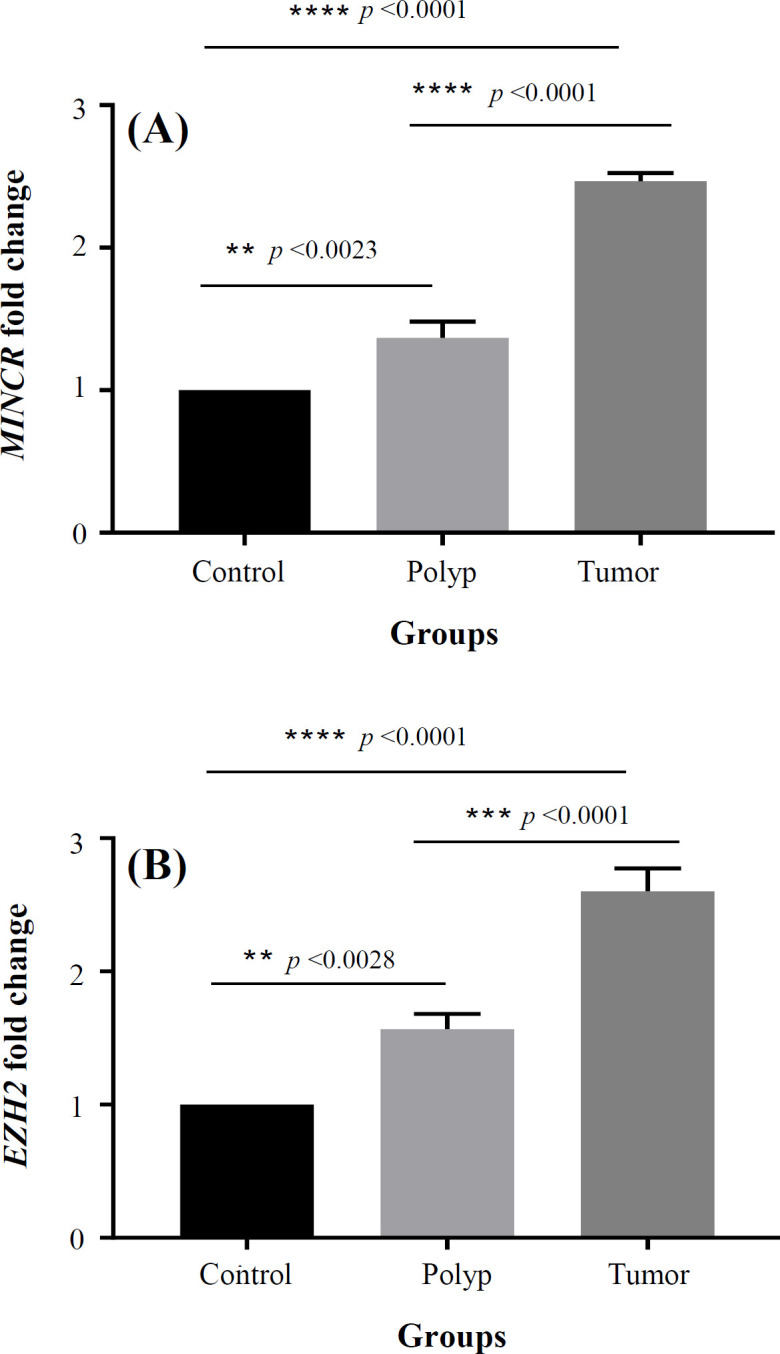
Expressions of *MINCR *(A) and *EZH2* (B) mRNA in polyp and tumor tissues compared to the control group (tumor-adjacent normal tissue). . (B) ^*^p < 0.05, ^**^p < 0.01, ^***^p < 0.001, and ^****^p < 0.0001

We determined the expression level of *MINCR* in CRC tissues. qRT-PCR analysis was performed in 43 tumor tissue samples, 36 polyp tissue samples, and 35 tumor adjacent normal tissue samples (control tissues). Results showed a higher expression level of *MINCR* in the tumor and polyp tissues in comparison to the neighboring normal tissues. According to the relative *MINCR* expression ratio (2.42) in the tumor tissue, *MINCR* demonstrated a significant upregulation in the CRC tissue as compared to the neighboring normal CRC tissues (Fig. 1A). We also investigated the association of the *MINCR* expression in the tumor tissue samples with clinicopathological characteristics. The results implied that the overexpression of *MINCR* had a significant association with the clinical stage (*p *= 0.0178) and type of samples (*p *= 0.0358), as represented in Table 3. Using real-time PCR, we evaluated the *EZH2* expression in CRC specimens. *EZH2* expression was significantly greater in the tumor and polyp tissues in comparison to adjacent normal tissues (Fig. 1B). The Fisher’s exact test results indicated a significant association of the high *EZH2* with the clinical stage (*p* = 0.0185) and type of samples (tumor and polyp tissues; *p* = 0.0478), as illustrated in Table 3.

## DISCUSSION

LncRNAs are unique molecules to function in tumorigenesis so that the aberrant and the dysregulation of their expression have been shown to be necessary in malignancy and tumor CRC expansion^[^^20^^,^^21^^]^. LncRNAs such as *MINCR* may essentially contribute to both *MYC*-positive lymphomas and numerous types of *MYC*-dependent cancers^[^^6^^]^. In this regard, the amplification of *MYC*-containing genomic region has been a common event in cancer and *MINCR* can mediate the effects of *MYC* overexpression on cancer progression^[^^22^^]^. In the present research, the expression level of *MINCR* in tumor and polyp tissue samples was evaluated. We first found that the *MINCR *was considerably overexpressed in both CRC and polyp tissues compared to the control group. In line with our data, Wang *et al.*’s^[^^13^^]^ study showed a positive correlation between the expression level of *MINCR* and TNM stage, larger tumor sizes, lymphatic metastasis, as well as shorter overall survival in gallbladder cancer.

*EZH2 *has been considered as one of the histone-lysine N-methyltransferase enzymes engaged in DNA methylation^[^^23^^]^. Moreover, its overexpression has been observed in the tumor tissues, but not normal ones^[^^24^^,^^25^^]^. Chen *et al.*^[^^26^^]^ have emphasized that the high expression of *EZH2* is related to tumor growth, metastasis, apoptosis suppression, and poor prognosis in cancer patients. The oncogenic contribution of *EZH2 *has shown in multiple kinds of the human cancers such as the breast and ovarian cancers^[^^27^^,^^28^^]^ so that it induces the expression of the tumor inhibitor genes ^[^^29^^]^. Some researchers have recently suggested *EZH2* a dual-faced molecule that could function both as a transcriptional repressor and an activator of post-translational alterations^[^^30^^,^^31^^]^. Our results showed that *EZH2* significantly increased in tumor and polyp tissues compared to the control group. This finding is in agreement with that of Boostani *et al.*^[^^32^^]^ who documented a significant association between *EZH2* and lymph node status. 

The upregulation of *MINCR* may participate in cancer initiation via triggering the *EZH2* expression. Systematically, findings have signified that *MINCR/EZH2* axis contributes to the rapid growth of the cells, cell invasion, and also apoptosis in the cancer cells. In addition, functional assays have revealed the suppression of the cell growth and G1/S arrest and the increase of the cell apoptosis through the *MINCR* knockdown. *MINCR* could stimulate the expression of *EZH2* via targeting miR-26a^[^^8^^]^. Therefore, MiR-26a has been considered as one of the essential regulators in tumorigenesis and cancer development and suppressors of tumor growth and metastasis^[^^7^^,^^10^^]^. Some studies have indicated that miR-26a may function as an oncogene in some cancer cells via the *AKT* pathway by targeting *PTEN* to promote cancer progression^[^^11^^,^^13^^]^. Yamamoto *et al.*^[^^33^^]^ have exhibited that *EZH2* expression is a prognostic biomarker in CRC patients treated with anti-epidermal growth factor receptor therapeutics. In our study, there had been an association between the high expression of *MINCR* and *EZH2* with the type of samples (tumor and polyp) and clinical stage.

**Table 3:** Correlation between the expression of *MINCR *and *EZH2* with clinicopathological characteristics

lncRNA/mRNA	*MINCR*		*EZH2*	
Characteristics	Number (%)	*p *value	Number (%)	*p *value
Clinical stage				
**II** **III**	14 (32.6) 29 (67.4)	0.0178	14 (32.6) 29 (67.4)	0.0185
Type of samples				
**Tumor** **Polyp**	43 (37.8) 36 (31.6)	0.0358	43 (37.8) 36 (31.6)	0.0478

According to the obtained information, our research is the first that evaluated the expression of lncRNA *MINCR *in CRC. We herein explored that *MINCR* may exert oncogenic effects in CRC and can be used as a potential biomarker for the detection of this cancer.

## CONFLICT OF INTEREST.

None declared.
